# Novel Benzothiazole, Benzimidazole and Benzoxazole Derivatives as Potential Antitumor Agents: Synthesis and Preliminary *in Vitro* Biological Evaluation

**DOI:** 10.3390/molecules17010873

**Published:** 2012-01-17

**Authors:** Pu Xiang, Tian Zhou, Liang Wang, Chang-Yan Sun, Jing Hu, Ying-Lan Zhao, Li Yang

**Affiliations:** State Key Laboratory of Biotherapy and Cancer Center, West China Hospital, West China Medicinal School, Sichuan University, Chengdu 610041, Sichuan, China

**Keywords:** benzothiazole derivatives, benzimidazole, benzoxazole, antitumor, cytosolic vacuolization

## Abstract

In a previous hit-to-lead research program targeting anticancer agents, two promising lead compounds, **1a** and **1b**, were found. However, the poor solubility of **1a** and **1b** made difficult further *in vivo* studies. To solve this problem, a lead optimization was conducted through introducing *N*-methyl-piperazine groups at the 2-position and 6-position. To our delight, the optimized analogue **1d** showed comparable antiproliferative activity *in vitro* with better solubility, compared with **1a**. Based on this result, the replacement of the benzothiazole scaffold with benzimidazole and benzoxazole moieties afforded **1f** and **1g**, whose activities were fundamentally retained. In the preliminary *in vitro* biological evaluation, the immunofluorescence staining of HCT116 cells indicated that **1d**, **1f** and **1g** led to cytosolic vacuolization which was not induced by **1a** at low micromolecular concentrations. These results suggest that these optimized compounds might potentially constitute a novel class of anticancer agents, which merit further studies.

## 1. Introduction

**Figure 1:**
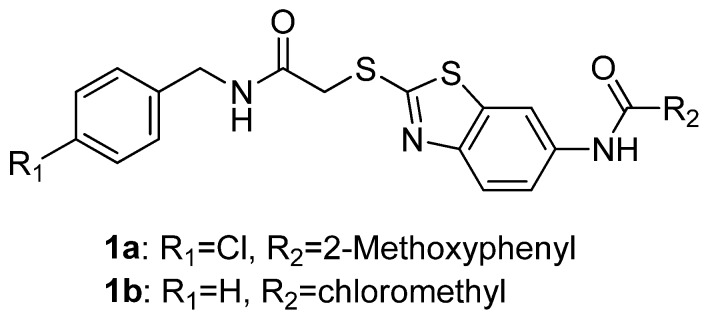
The structure of lead compounds **1a** and **1b**.

Cancer is the leading cause of death in developed countries and the second leading cause of death in developing countries [1]. Although surgical resection is potentially curative, the risk of recurrence remains very high. Besides surgical resection, treatment strategies for high risk patients are still mainly based on adjuvant or neoadjuvant use of chemotherapeutic agents alone or in combination with radiotherapy. Unfortunately, the use of the above standard therapeutic protocols only results in a moderate decline in mortality and the risk of sustaining a recurrence of disease remains high. Thus, it is urgent to develop novel chemotherapeutic agents for the treatment of cancer. In a recent hit-to-lead research of anticancer agents [2], lead compounds **1a** and **1b** (Figure 1) with benzothiazole scaffolds, showed promising anticancer *in vitro* activities. However, the solubility of **1a** and **1b** was far from satisfactory for further *in vivo* research. As a result, a lead optimization of **1a** and **1b** was conducted.

**Figure 2:**
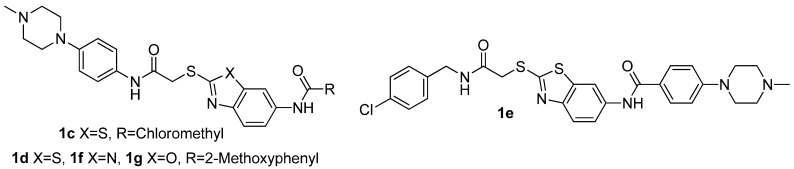
The structures of compounds **1c–g**.

The *N*-methyl-piperazine group as found in imatinib is a commonly used motif in lead optimization [3]. Introduction of *N*-methylpiperazine moieties at the 2-position and 6-positions of the benzothiazole scaffold afforded compounds **1c**, **1d** and **1e**. To determine the role of the benzothiazole scaffold, the benzoxazole analogue **1f** and benzimidazole analogue **1g** were also designed (Figure 2).

Herein, we report the synthesis of **1c–g**, preliminary structure-activity relationships (SARs) and biological evaluation of these anticancer agents.

## 2. Results and Discussion

### 2.1. Chemistry

**Scheme 1:**
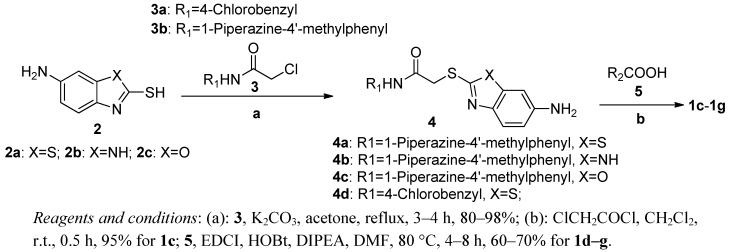
General procedures for the synthesis of **1c–g**.

The synthetic route of compounds **1c–g** using a modified literature method [4,5] is outlined in Scheme 1. Taking **1d** for example, the key intermediate **2a**, 2-mercapto-6-aminobenzothiazole, was alkylated with side chain **3b** under basic conditons to give **4a**. Subsequent acylation of **4a** with 2-methoxybenzoic acid yielded the target compound **1d**. Besides the optimization of side chains, the replacement of the benzothiazole scaffold with benzimidazole and benzoxazole units (Table 1), afforded **1f** and **1g** which were prepared using the same synthetic strategy.

**Table 1:** The anti-proliferative activity of **1a–g** against HepG2 and HCT-116 cells.
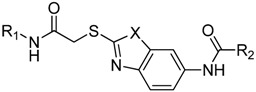

Compound	R_1_	R_2_	X	IC_50_(μM) ^a^	
				HepG2	HCT-116
**1a**	4-Chlorobenzyl	2-Methoxyphenyl	S	0.64	1.52
**1b**	Benzyl	Chloromethyl	S	1.13	1.16
**1c**	*N*-methyl-piperazine-phenyl	Chloromethyl	S	>40.0	>40.0
**1d**	*N*-methyl-piperazine-phenyl	2-Methoxyphenyl	S	2.10	1.25
**1e**	4-Chlorobenzyl	*N*-methylpiperazine-phenyl	S	31.2	28.8
**1f**	*N*-methyl-piperazine-phenyl	2-Methoxyphenyl	N	14.6	3.69
**1g**	*N*-methyl-piperazine-phenyl	2-Methoxyphenyl	O	12.2	2.61

^a^ The cytotoxicity effects of various compounds on cancer cells were determined by the MTT assay, and the results were expressed as the mean IC_50_ calculated from three independent experiments.

**Scheme 2:**
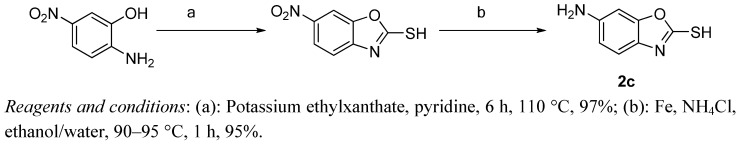
Synthesis of 6-aminobenzofuran-2-thiol **2c**.

The key intermediate **2c** was readily prepared from commercially available 2-amino-5-nitrophenol following cyclization and subsequent reduction of the nitro group according to the method represented in Scheme 2.

**Scheme 3:**
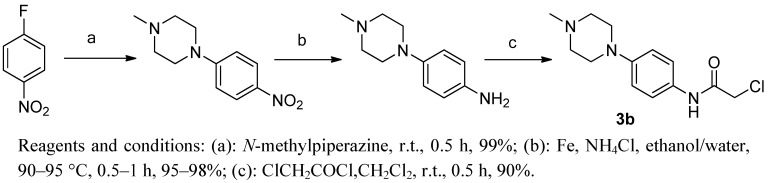
Synthesis of 2-chloro-*N*-(4-(4-methylpiperazin-1-yl)phenyl)acetamide **3b**.

Side chain **3b** was prepared from 4*-*fluoronitrobenzene condensed with *N*-methylpiperazine, subsequent reduction and acylation as shown in Scheme 3.

### 2.2. The MTT Assay

With **1c–g** at hand, we evaluated their anti-proliferative activities (IC_50_) on HepG2 and HCT116 cells, using the MTT assay (Table 1). As shown in the Table, introduction of an *N*-methylpiperazine substituent at the 2-position (compound **1d**) in the R1 group was well tolerated. To our surprise, replacement of the benzyl group in R1 with an *N*-methylpiperazine-phenyl group (compound **1c**) led to almost total loss of activity. The introduction of *N*-methylpiperazine substituent at the 6-position (**1e**) in the R2 group had a detrimental effect on the potency, that is, about 20–50-fold decrease in potency, compared with that of **1a**. After identifying that the *N*-methylpiperazine-phenyl substituent is well tolerated at the 2-position in the R1 group, we next turned to examining the role of the benzothiazole scaffold. Replacement of the benzothiazole ring with benzimidazole and benzoxazole yielded **1f** and **1g** with a slightly decrease in potency for HCT-116 cells compared with **1a**, but showed no clear SAR trends. It was noteworthy that analogue **1d** exhibited comparable inhibitory activity against HepG2 and HCT-116 cells.

To further study the cytotoxic profile, compound **1d** was selected for evaluation of inhibitory activities against a panel of different types of human cancer cell lines: Prostate cancer cell line PC-3, human cervical carcinoma cell line Hela, ovarian cancer cell line SKOV-3, lung cancer cell line A549, melanoma cell line A375, nonsmall cell lung cancer cell line H460 and epithelial cancer cell line A431 (Table 2). As shown in Table 2, compound **1d** also showed broad-spectrum inhibitory activity which was exhibited in previous research [2]. Some IC_50_ values were better than **1a**, such as against Hela and SKOV-3 cells.

**Table 2:** The anti-proliferative activities of **1d** against various cancer cell lines.

Compound	IC_50_(μM) ^a^						
	PC-3	Hela	SKOV-3	A549	A375	H460	A431
**1a**	1.3	4.5	6.2	1.9	2.2	2.3	1.3
**1d**	1.2	1.5	1.3	1.1	2.3	1.8	2.5

^a^ The cytotoxicity effects of compounds on cancer cells were determined by the MTT assay, and the results were expressed as the mean IC_50_ calculated from three independent experiments.

### 2.3. The Solubility Assay

The optimized analogues **1d**, **1f** and **1g** showed comparable anti-proliferative activities *in vitro* compared with **1a**. To conduct a preliminary assessment of the absorption *in vivo*, solubility was further determined (Table 3). As shown in Table 3, **1d**, **1f** and **1g** showed better solubility both in ethanol and water after the introduction of *N*-methylpiperazine group.

**Table 3:** The solubility of compound **1a**, **1d**, **1f** and **1g**.

Solvent (μg/mL)	1a	1d	1f	1g
ethanol	1.77	317.9	2803	572.2
water	0.093	5.99	981.7	548.4

### 2.4. Immunofluorescence Staining

**Figure 3:**
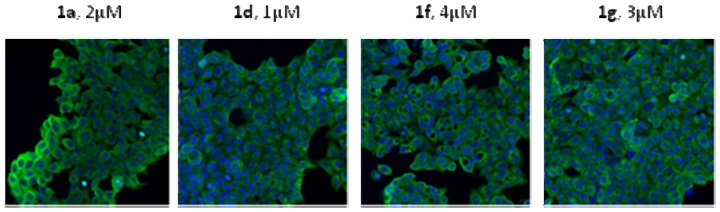
The immunofluorescence staining of **1a**, **1d**, **1f** and **1g** on HCT-116 cells for 24 h. Blue: nuclear; Green: tubulin.

In our previous research, it was found that **1a** could induce apoptosis of cancer cells. To further study the antitumor mechanisms of **1a**, **1d**, **1f** and **1g** *in vitro*, an immunofluorescence staining of HCT116 cells was conducted (Figure 3).

As shown in Figure 3, the tubulin of HCT-116 cells was intact after the treatment with 1d, 1f and 1g. However, cytosolic vacuolization appeared, which was’t observed for 1a. It has been reported that this kind of cytosolic vacuolization is related with the inhibition of HSP-90 protein or the proteasome-promoted protein ubiquitination [6]. Further mechanistic studies are currently in progress.

## 3. Experimental

### 3.1. General

The human cancer cell lines, HepG2, HCT116, PC-9, Hela, SKOV-3, A549, A375, H460 and A431 were purchased from the American Type Culture Collection (ATCC, Rockville, MD, USA). Dulbecco’s modified Eagle medium (DMEM) and RPMI 1640 were purchased from Gibco (Grand Island, NY, USA). Fetal bovine serum (FBS) was obtained from Hyclone (Logan, UT, USA). The purity of compound screened in biological assays was determined to be >98% by HPLC analysis. An Symmetry C_18_ (75 mm × 4.6 mm, i.d. 3.5μm) (Waters, Milford, MA, USA) was used with a t elution of acetonitrile and HPLC-grade water as mobile phase. ^1^H-NMR were recorded at 400 MHz on a Varian spectrometer (Varian, Palo Alto, CA, USA) model Gemini 400. Mass Spectra (MS) were measured by Q-TOF Priemier mass spectrometer utilizing electrospray ionization (ESI) (Micromass, Manchester, UK).

### 3.2. Preparation of 6-Aminobenzo[d]oxazole-2-thiol (**2c**)

#### 3.2.1. 6-Nitrobenzo[d]oxazole-2-thiol

6-Nitrobenzo[d]oxazole-2-thiol was synthesized according to a modified literature method [7]. A suspension of 2-amino-5-nitrophenol (3.08 g, 20 mmol) and potassium ethylxanthate (3.36 g, 21 mmol) in dry pyridine (40 mL) was stirred at 120 °C for 6 h, and then at room temperature for another 16 h. 2 M HCl solution was added to the solution to adjust the pH value to 6. The resulting precipitate was collected by filtration, washed with petroleum ether and then dried in vacuum to afford 3.22 g (97%) of the title compound. ^1^H-NMR (DMSO-*d_6_*) δ: 7.39 (d, 1H, *J* = 8 Hz), 7.86 (t, 1H, *J* = 8 Hz), 8.22 (dd, 1H, *J_1_* = 4 Hz, *J_2_* = 8 Hz)), 8.41 (s, 1H). ESI-MS (*m/z*, %): 197.1 (M+H)^+^.

#### 3.2.2. 6-Aminobenzo[d]oxazole-2-thiol (**2c**)

A mixture of 6-nitrobenzo[d]oxazole-2-thiol (5 g, 25 mmol), iron powder (3.36 g, 60 mmol), and ammonium chloride (2.67 g, 50 mmol) was refluxed in a mixed solvent of ethanol (25 mL) and H_2_O (8 mL). After completion of the reaction, the mixture was filtered while hot and washed with petroleum ether. Standing at room temperature gave compound **2c** as a bright yellow solid. Yield: 3.7 g, 90%. ^1^H-NMR (DMSO-*d_6_*) δ: 6.51 (dd, 2H, *J_1_* = 4 Hz, *J_2_* = 8 Hz), 6.64 (d, 2H, *J* = 4 Hz), 6.90 (d, 2H, *J* = 8 Hz). ESI-MS (*m/z*, %): 167.1 (M+H)^+^.

### 3.3. 2-Chloro-N-(4-chlorobenzyl)acetamide (**3a**)

2-Chloroacetyl chloride (4.79 g, 42 mmol) was added dropwise with stirring to a mixture of (4-chlorophenyl) methanamine (4.96 g, 35 mmol) in dichloromethane (50 mL) cooled to 0 °C. The resulting mixture was allowed to warm to ambient temperature and stirred for 1 h. After filtering, the solid was washed with petroleum ether and dried under vacuum for 12 h at 25–30 °C. The title compound **3a** was obtained by recrystalization from ethanol. Yield: 7.25 g, 95%.^1^H-NMR (400 MHz, DMSO-*d*_6_): δ 4.13 (s, 2H), 4.29 (d, *J* = 6.0 Hz, 2H), 7.28 (d, *J* = 4.4 Hz, 2H), 7.39 (d, *J* = 8.0 Hz, 2H), 8.77 (s, 1H). ESI-MS (*m/z*, %): 218.0 (M−H)^+^.

### 3.4. 2-Chloro-*N*-(4-(4-methylpiperazin-1-yl)phenyl)acetamide (**3b**)

#### 3.4.1. 1-Methyl-4-(4-nitrophenyl)piperazine

Briefly, 1-methylpiperazine (15.6 g, 156 mmol) was added slowly while stirring to a solution of 4-fluoronitrobenzene (20 g, 142 mmol) at room temperature. The mixture was stirred for 1 h, then filtered and washed with petroleum ether to give product as an orange solid. Yield: 30.3 g, 96.43%.^1^H-NMR (DMSO-*d_6_*) δ: 2.22 (s, 3H), 2.43 (t, 4H, *J* = 4 Hz), 3.45 (t, 4H, *J* = 4 Hz), 7.03 (d, 2H, *J* = 8 Hz), 8.05 (d, 2H, *J* = 8 Hz). ESI-MS (*m/z*, %): 222.0 (M+H)^+^.

#### 3.4.2. 4-(4-Methylpiperazin-1-yl)aniline

A mixture of 1-methyl-4-(4-nitrophenyl) piperazine (14.8 g, 67 mmol), iron powder (15 g, 268 mmol), ammonium chloride (7.2 g, 134 mmol) was refluxed in a mixed solvent of ethanol (60 mL) and H_2_O (20 mL). After completion of the reaction, the mixture was filtered while hot and washed with petroleum ether. After standing at room temperature an off-white acicular solid was obtained. Yield: 12.2 g, 95.31%. ^1^H-NMR (DMSO-*d_6_*) δ: 2.22 (s, 3H), 2.5 (t, 4H, *J* = 4 Hz), 2.75 (t, 4H, *J* = 4 Hz), 3.22 (s, 2H), 6.55 (d, 2H, *J* = 8 Hz), 6.74 (d, 2H, *J* = 8 Hz). ESI-MS (*m/z*, %): 192.0 (M+H)^+^.

#### 3.4.3. 2-Chloro-N-(4-(4-methylpiperazin-1-yl)phenyl)acetamide (**3b**)

2-Chloroacetyl chloride (4.79 g, 42 mmol) was added dropwise with stirring to a mixture of 1-methyl-4-(4-nitrophenyl) piperazine (6.69 g, 35 mmol) in dichloromethane (50 mL) cooled at 0 °C. The resulting mixture was allowed to warm to ambient temperature and stirred for 1 h. After filtering, the solid was washed with petroleum ether and dried under vacuum for 12 h at 25–30 °C. The title compound **3b** was obtained by recrystalization from a solution of ethanol. Yield: 8.48 g, 90.51%.^1^H-NMR (DMSO-*d_6_*) δ: 2.21 (s, 3H), 2.43 (t, 4H, *J* = 4.8 Hz), 3.07 (t, 4H, *J* = 4.8 Hz), 4.2 (s, 2H), 6.90 (d, 2H, *J* = 8 Hz), 7.42 (d, 2H, *J* = 8 Hz), 10.10 (s, 1H). ESI-MS (*m/z*, %): 268.1 (M+H)^+^.

### 3.5. General Procedure for Preparing Compounds **4a–d**

Compound **3a–b** (13.76 mmol) was added to a mixture of **2a–c** (16.51 mmol) and potassium carbonate (20.64 mmol) in acetone (25 mL) while refluxing with stirring. The resulting mixture was stirred under reflux for 4 h, then cooled to ambient temperature. After removal of acetone on a rotary evaporator, the solid was dissolved in ethyl acetate (100 mL) and washed with water (2 × 10 mL), saturated solution of sodium bicarbonate (2 × 20 mL) and sodium chloride (2 × 20 mL). Then, the organic phase was dried over Na_2_SO_4_ and concentrated *in vacuo* to give a solid product. The title compounds **4a–d** were thus obtained, and could be used directly for the next step without further puriﬁcation. The yields of **4a**, **4b**, **4c** and **4d**, were 92%, 90%, 90% and 93%, respectively.

*2-(6-aminobenzo[d]thiazol-2-ylthio)-*N*-(4-(4-methylpiperazin-1-yl)phenyl)acetamide* (**4a**): ^1^H-NMR (DMSO-*d_6_*) δ: 2.24 (s, 3H), 2.44 (t, 4H, *J* = 4 Hz), 3.06 (t, 4H, *J* = 4 Hz), 4.14 (s, 2H), 5.38 (s, 2H), 6.70 (dd, 1H, *J_1_* = 2 Hz, *J_2_* = 8.8 Hz), 6.88 (d, 2H, *J* = 9.2 Hz), 7.00 (d, 1H, *J* = 2.4 Hz), 7.41 (d, 2H, *J* = 8.8 Hz), 7.49 (d, 1H, *J* = 8.8 Hz), 10.16 (s, 1H). ESI-MS (*m/z*, %): 414.2 (M+H)^+^.

*2-(6-amino-1H-benzo[d]imidazol-2-ylthio)-*N*-(4-(4-methylpiperazin-1-yl)phenyl)acetamide* (**4b**): ^1^H-NMR (DMSO-*d_6_*) δ: 2.23 (s, 3H), 2.47 (s, 4H), 3.07 (s, 4H), 4.21 (s, 2H), 5.31 (s, 2H), 6.46 (d, 1H, *J* = 4 Hz), 6.56 (s, 1H), 6.88 (d, 2H, *J* = 8.8 Hz), 7.16 (s, 1H), 7.42 (d, 2H, *J* = 8.8 Hz), 10.40 (s, 1H), 12.13 (s, 1H). ESI-MS (*m/z*, %): 397.3 (M+H)^+^.

*2-(6-aminobenzo[d]oxazol-2-ylthio)-*N*-(4-(4-methylpiperazin-1-yl)phenyl)acetamide* (**4c**): ^1^H-NMR (DMSO-*d_6_*) δ: 2.23 (s, 3H), 2.46 (t, 4H, *J* = 4 Hz), 3.07 (t, 4H, *J* = 4 Hz), 4.23 (s, 2H), 5.31 (s, 2H), 6.56 (dd, 1H, *J_1_* = 2 Hz, *J_2_* = 8.4 Hz), 6.71 (d, 1H, *J* = 1.6 Hz), 6.89 (d, 2H, *J* = 8 Hz), 7.22 (t, 1H, *J* = 8 Hz), 7.41 (d, 2H, *J* = 8 Hz), 10.16 (s, 1H). ESI-MS (*m/z*, %): 398.2 (M+H)^+^.

*2-(6-aminobenzo[d]thiazol-2-ylthio)-N-(4-chlorobenzyl)acetamide* (**4d**): ^1^H-NMR (DMSO-*d_6_*) δ: 4.09 (s, 2H), 4.30 (d, 2H, *J* = 5.6 Hz), 5.40 (s, 2H), 6.73 (dd, 1H, *J_1_* = 1.6 Hz, *J_2_* = 8.8 Hz), 7.01 (d, 1H, *J* = 1.6 Hz), 7.28 (q, 4H, *J* = 8.4 Hz), 7.49 (d, 1H, *J* = 8.4 Hz), 8.81 (t, 1H, *J* = 5.6 Hz). ESI-MS (*m/z*, %): 364.0 (M+H)^+^.

### 3.6. 2-Chloro-N-(2-(2-(4-(4-methylpiperazin-1-yl)phenylamino)-2-oxoethylthio)benzothiazol-6-yl)-acetamide (**1c**)

2-Chloroacetyl chloride (43 mg, 0.38 mmol) was added dropwise with stirring to a solution of **4a** (80 mg, 0.19 mmol) in dichloromethane (4 mL) originally at 0 °C. The resulting mixture was allowed to warm to ambient temperature and stirred for 1 h. After filtering, the solid was washed with petroleum ether and dried under vacuum for 12 h at 25–30 °C. The title compound **1c** (an off-white solid) were obtained by recrystallization from ethanol. Yield: 72 mg, 77.42%. m.p. 245.8–246.1 °C. ^1^H-NMR (DMSO-*d_6_*) δ: 2.21 (s, 3H), 2.43 (t, *J* = 4.4 Hz, 4H), 3.07 (t, 4H, *J* = 4.8 Hz), 4.30 (s, 2H), 4.35 (s, 2H), 6.93 (d, 2H, *J* = 8.8Hz), 7.49 (d, 2H, *J* = 8.8 Hz), 7.55 (dd, 1H, *J* = 2 Hz, 8.8 Hz), 7.79 (d, 1H, *J* = 8.8 Hz), 8.39 (d, 1H, *J* = 2 Hz), 10.32 (s, 1H), 10.62 (s, 1H); ESI-MS (*m/z*, %): 490.3 (M+H)^+^.

### 3.7. General Procedure for Preparing Compounds **1d–g**

A mixture of compound **4a–d** (0.5 mmol), 2-methoxybenzoic acid hydrate or 4-(4-methylpiperazin-1-yl)benzoic acid (0.75 mmol), EDCI (0.75 mmol), HOBt (0.75 mmol), DIPEA (0.75 mmol) was warmed up to 80 °C in DMF (5 mL) with stirring. After completion of reaction, the mixture was diluted with ethyl acetate (100 mL). The resulting mixture was washed with washed with water (2 × 20 mL), saturated solution of sodium bicarbonate (2 × 20 mL) and sodium chloride (2 × 20 mL). Then, the organic phase was dried over Na_2_SO_4_ and concentrated *in vacuo* to give a solid product. The title compounds were obtained by recrystallization from ethanol or ethyl acetate.

*2-Methoxy-*N*-(2-(2-(4-(4-methylpiperazin-1-yl)phenylamino)-2-oxoethylthio)benzo[d]thiazol-6-yl)benzamide* (**1d**). Off-white solid. Yield: 76 mg, 75.47%. m.p. 172.1–172.4 °C. ^1^H-NMR (DMSO-*d_6_*) δ: 2.10 (s, 3H), 2.22 (t, 4H, *J* = 4.4 Hz), 3.08 (t, 4H, *J* = 4.8 Hz), 3.91 (s, 3H), 4.35 (s, 3H), 6.91 (d, 2H, *J* = 8.8 Hz), 7.09 (t, 1H, *J* = 7.2 Hz), 7.20 (d, 1H, *J* = 8.4 Hz), 7.48 (d, 2H, *J* = 8.4 Hz), 7.53 (t, 1H, *J* = 8 Hz), 7.67 (q, 2H, *J* = 8.8 Hz), 7.80 (d, 1H, *J* = 8.4 Hz), 8.55 (s, 1H), 10.22 (s,1H), 10.36 (s,1H); ESI-MS (*m/z*, %): 548.0 (M+H)^+^.

N*-(2-(2-(4-Chlorobenzylamino)-2-oxoethylthio)benzo[d]thiazol-6-yl)-4-(4-methylpiperazin-1-yl)-benzamide* (**1e**). Yellow solid. Yield: 184 mg, 65.36%. m.p. 177.8–179.2 °C. ^1^H-NMR (DMSO-*d_6_*) δ: 2.09 (s, 3H), 2.45 (t, 4H, *J* = 4.4 Hz), 3.30 (t, 4H, *J* = 4.8 Hz), 4.19 (s, 2H), 4.31 (d, 2H, *J* = 6 Hz), 7.03 (d, 2H, *J* = 8.8 Hz), 7.28 (d, 2H, *J* = 8.8 Hz), 7.32 (d, 2H, *J* = 8.4 Hz), 7.76 (s, 2H), 7.89 (d, 2H, *J* = 8.8 Hz), 8.52 (s, 1H), 8.85 (t, 1H, *J* = 6Hz), 10.17 (s, 1H); ESI-MS (*m/z*, %): 566.2 (M+H)^+^.

*2-Methoxy-*N*-(2-(2-(4-(4-methylpiperazin-1-yl)phenylamino)-2-oxoethylthio)-1H-benzo[d]imidazol-6-yl)benzamide* (**1f**). Faint yellow solid. Yield: 90 mg, 81.08%. m.p. 132.3–132.6 °C. ^1^H-NMR (DMSO-*d_6_*) δ: 2.21 (s, 3H), 2.43 (t, 4H, *J* = 4 Hz), 3.06 (t, 4H, *J* = 4 Hz), 3.90 (s, 3H), 4.21 (s, 2H), 6.88 (d, 2H, *J* = 8.8 Hz), 7.07 (t, 1H, *J* = 7.2 Hz), 7.18 (d, 1H, *J* = 8 Hz), 7.30 (q, 1H, *J* = 8.4 Hz), 7.43 (t, 3H, *J* = 8.8 Hz), 7.50 (t, 1H, *J* = 7.6 Hz), 7.64 (d, 1H, *J* = 7.2 Hz), 8.07 (d, 1H, *J* = 33.2 Hz), 10.08 (d, 1H, *J* = 32.8 Hz), 10.27 (s, 1H), 12.60 (s, 1H); ESI-MS (*m/z*, %): 531.3 (M+H)^+^.

*2-Methoxy-*N*-(2-(2-(4-(4-methylpiperazin-1-yl)phenylamino)-2-oxoethylthio)benzo[d]oxazol-6-yl)-benzamide* (**1g**). Yellow solid. Yield: 100 mg, 75.75%. m.p. 210.4–210.8 °C. ^1^H-NMR (DMSO-*d_6_*) δ: 2.24 (s, 3H), 2.40 (t, 4H, *J* = 4.4 Hz), 3.14 (t, 4H, *J* = 4.8 Hz), 3.89 (s, 3H), 4.23 (s, 2H), 6.91 (d, 2H, *J* = 8.8 Hz), 7.09 (t, 1H, *J* = 7.2 Hz), 7.20 (d, 1H, *J* = 8.4 Hz), 7.48 (d, 2H, *J* = 8.4 Hz), 7.53 (t, 1H, *J* = 8 Hz), 7.67 (q, 2H, *J* = 8.8 Hz), 7.80 (d, 1H, *J* = 8.4 Hz), 8.26 (s, 1H), 9.98 (s,1H), 10.29 (s,1H); ESI-MS (*m/z*, %): 532.2 (M+H)^+^.

### 3.8. Cell Culture

Cell lines including HepG2, Hela, Skov-3, A375 and A431 were maintained in Dulbecco’s modified Eagle medium (DMEM) containing 10% fetal bovine serum (FBS), penicillin (100 U/mL) and streptomycin (10 mg/L). Cell lines including HCT116, PC-9, A549 and H460 were maintained in RPMI 1640 containing 10% fetal bovine serum (FBS), penicillin (100 U/mL) and streptomycin (10 mg/L). Cells were grown in a 5% CO_2_ incubator at 37 °C.

### 3.9. Cell Proliferation Assay (MTT Assay)

Cells (2 × 10^3^/well) were seeded in 96-well plates and cultured for 24 h, followed by compounds which dissolved in dimethylsulfoxide (DMSO) treatment for 48 h. Ten microliters of 10 mg/mL MTT was added and cultured for 4 h, the medium was removed and 150 μL of DMSO was added to dissolve formazan crystals. Absorbance was measured at 570 nm using an SpectraMAX M5 microplate spectrophotometer (Molecular Devices). The proliferation inhibitory effects of the compounds on cancer cells were expressed as IC_50_.

### 3.10. Immunofluorescence

Cells (1 ×10^5^) were plated in 6-well plates. Following drug treatment for 24 h, cells were washed with PBS, punched by Triton X-100. Subsequently cells were probed by the primary antibodies. Then cells were incubated with the secondary antibody for 30 min at room temperature and examined under a fluorescence microscope [8].

## 4. Conclusions

In this paper, five novel benzothiazole-2-thiol, benzimidazole-2-thiol and benzoxazole-2-thiol derivatives were synthesized for lead optimization, and their *in vitro* antitumor activity was evaluated. To our delight, compounds **1d**, **1f** and **1g** showed comparable antitumor activities and better solubility compared with the lead compound **1a**. In a preliminary antitumor activity mechanism study, we found a novel phenomenon, that is, cytosolic vacuolization after treatment with these compounds which was not induced by **1a**. Thus, it indicates that compounds **1d**, **1f** and **1g** might possess different anticancer mechanism, which deserves further study.
